# The Genetic Diversity and Evolution of HIV-1 Subtype B Epidemic in Puerto Rico

**DOI:** 10.3390/ijerph13010055

**Published:** 2015-12-23

**Authors:** Pablo López, Vanessa Rivera-Amill, Nayra Rodríguez, Freddie Vargas, Yasuhiro Yamamura

**Affiliations:** AIDS Research Program, Ponce Health Sciences University-School of Medicine/Ponce Research Institute, 395 Industrial Reparada 2, Ponce, PR 00716-2348, USA; plopez@psm.edu (P.L.); vrivera@psm.edu (V.R.-A.); nrodriguez@psm.edu (N.R.); fvargas@psm.edu (F.V.)

**Keywords:** HIV-1 subtype B, Puerto Rico, genetic diversity, evolution

## Abstract

HIV-1 epidemics in Caribbean countries, including Puerto Rico, have been reported to be almost exclusively associated with the subtype B virus (HIV-1B). However, while HIV infections associated with other clades have been only sporadically reported, no organized data exist to accurately assess the prevalence of non-subtype B HIV-1 infection. We analyzed the nucleotide sequence data of the HIV *pol* gene associated with HIV isolates from Puerto Rican patients. The sequences (*n* = 945) were obtained from our “HIV Genotyping” test file, which has been generated over a period of 14 years (2001–2014). REGA subtyping tool found the following subtypes: B (90%), B-like (3%), B/D recombinant (6%), and D/B recombinant (0.6%). Though there were fewer cases, the following subtypes were also found (in the given proportions): A1B (0.3%), BF1 (0.2%), subtype A (01-AE) (0.1%), subtype A (A2) (0.1%), subtype F (12BF) (0.1%), CRF-39 BF-like (0.1%), and others (0.1%). Some of the recombinants were identified as early as 2001. Although the HIV epidemic in Puerto Rico is primarily associated with HIV-1B virus, our analysis uncovered the presence of other subtypes. There was no indication of subtype C, which has been predominantly associated with heterosexual transmission in other parts of the world.

## 1. Introduction

The human immunodeficiency virus (HIV) is characterized by its high genetic variability as a result of its high replication rate, large progeny size, and short generation time, all of which are coupled with the low fidelity of its reverse transcriptase [1,2]. A consequence of this variability and subsequent high rate of evolution is the potential impact of those characteristics in terms of the efficacy of a future vaccine and their possible influence on progression, infectivity, and transmissibility as well as on a given infected individual’s response to anti-retroviral treatment. Thus, the sequences of the virus have been classified on the basis of their phylogenetic relationships so that virus evolution can be more easily monitored [3]. On the Caribbean island of Puerto Rico (a US commonwealth), approximately 47,000 individuals have been diagnosed with HIV/AIDS as of June 2015 (HIV/AIDS Surveillance Program, Office of Epidemiology and Research, PR Health Department). According to the Center of Disease Control and Prevention (CDC) HIV surveillance report of 2012, the HIV incidence rate in Puerto Rico was estimated at 24.2 cases per 100,000 population. That rate is approximately 40% higher than the national average for the US, including all the states and territories.

The HIV-1 subtype distribution analysis, as reported in the geography interface of the Los Alamos database [4], indicates that the HIV-1 subtype B (HIV-1B) is the most common variant and represents 99.5% of the total infections. HIV-1B is a variant that is part of the large group of subtypes that is contained within group M and that is predominant in North America, Western Europe, and the Caribbean [5]. Nevertheless, other sub-types, such as subtype F and several different circulating recombinant forms (CRFs), regularly introduce new variables that should be monitored; however, very little is known about the genetic diversity and evolutionary history of HIV-1 in Puerto Rico [6].

## 2. Methods

The current study was conducted in accordance with the Declaration of Helsinki, and the protocol was certified by the Institutional Review Board of the Ponce Research Institute to be exempt from the federal policy for the protection of human subjects under the provision of use of existing data and specimens (Protocol number: 150504-YY; date of approval: 5 June 2015). We analyzed the nucleotide sequences data on HIV *pol* gene associated with HIV Puerto Rican isolates. Sequences (*n* = 945) were obtained from our “HIV Genotyping” test-file covering a period of 14 years (2001–2014). Demographic data shows that the sample was predominantly male (67%) and the mean ages for men and women were 42 (4–76) and 41 (5–77) years, respectively. No risk factor information was available. Sequences were from antiretroviral-treated subjects and phylogenetically analyzed using the Maximum Likelihood Method based on the Tamura-Nei model in MEGA6 [7] to verify that all sequences were derived from different subjects. (Figure S1). Positions known to be associated with drug resistance mutations were excluded to avoid bias during the analyses [8,9]. The sequences were processed by TRUGENE HIV-1 Genotyping Assay (Siemens) protocols, according to the manufacturer’s instructions. Sequences were aligned and edited using BioEdit software (v7.0.5.2) and HIV subtype were determined using the REGA subtyping tool (v3) [10]. Bayesian analysis was performed by using Bayesian Evolutionary Analysis by Sampling Trees (BEAST) software v1.4.8 [11] to estimate the timescale of the epidemic and demographic growth patterns (B/D recombination samples). We considered the non-parametric Bayesian Skyline Plot (BSP) coalescent prior to infer the demographic history, which included a relaxed molecular clock to measure the evolutionary change over time in our samples and a gamma-distributed rate variation [12,13,14,15]. Coalescent analysis was constructed under general time-reversible model (GTR) suggested by Modeltest [4,16]. The Bayesian calculation consisted of 200,000,000 generations of Markov Chains Monte Carlo (MCMC) with a sampling done each 10,000 generations in order to achieve an Effective Sampling Size (ESS). To summarize the posterior distribution, a consensus tree was generated (TreeAnnotator v.5.5.4) discarding the first 10% as burn-in. BSP plot was generated using Tracer v1.5.0 [17]. The sequences were submitted to Gen Bank with accession numbers: KT374385-KT375039 and KT385879-KT386294.

## 3. Results and Discussion

In the present study, we evaluated the genetic diversity of the HIV-1 virus and the evolution of B/D recombinants in Puerto Rico. Because of the high level of travel between the US and Puerto Rico, how the virus evolved has become an important issue that needs to be analyzed. Global HIV epidemics involve highly diverse clades of the virus. In contrast, HIV epidemics found in different Caribbean countries, including Puerto Rico, have been reported to be almost exclusively associated with HIV-1 subtype B virus, and HIV infections associated with other clades (including F-clade and CRFs) have been only sporadically reported. Currently, no organized database that can be used in the accurate assessment of the prevalence of HIV infection by non–B-clade recombinant viruses exists. Because of what is mentioned above, studies of the HIV-1 virus on the island are focused primarily on subtype B.

**Figure 1:**
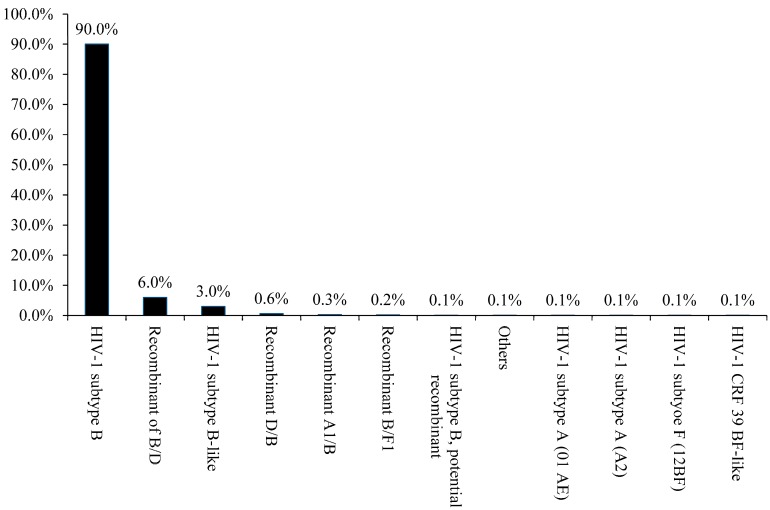
HIV-1 Subtype B is the predominant form in Puerto Rico (2001–2014). A total of 945 sequences were analyzed and the diagram was created by using Krona visualization tool [26].

**Figure 2:**
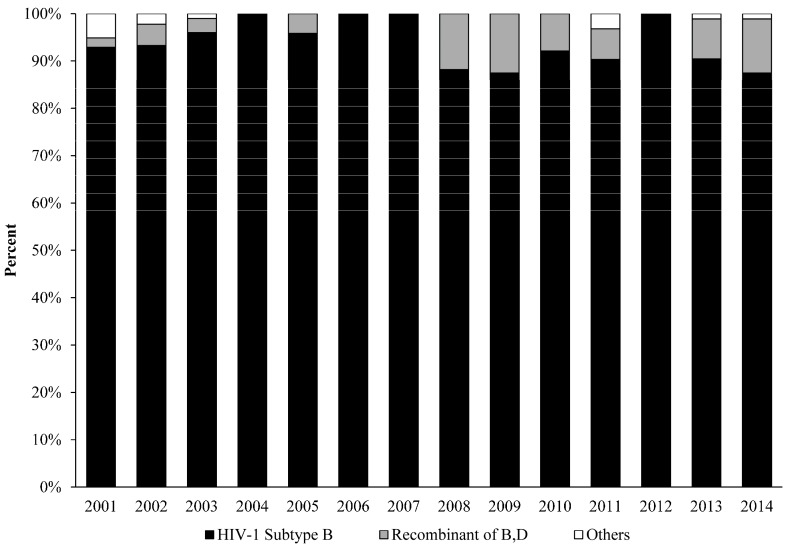
REGA (version 3) sub-typing analysis revealed a high prevalence of HIV-1 B-clade and recombinants, including subtype B (92.8%) and B/D recombinant (5.9%). A much smaller number of cases also involved (1.3%); subtype A (A1), A (01-AE), A (A2-like), CRF-39 BF-like, recombinant 39-BF/D, and others. Some of the recombinants appeared as early as in 2001.

**Figure 3:**
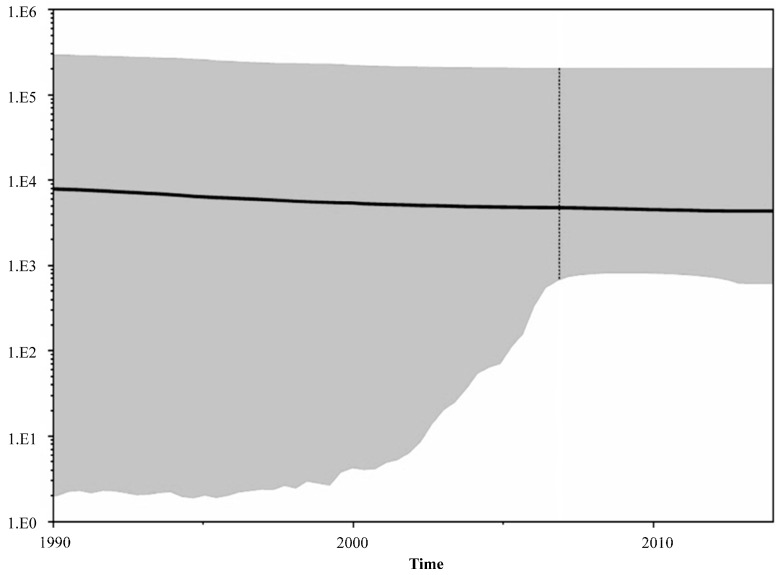
Bayesian skyline plots for the HIV-1 recombinant B/D in Puerto Rico (2011–2014). (*n* = 40) The plots represent number of effective population size (*Y* = log10) through time (*X* = time). The line represents the median of number of lineages with upper and lower 95% highest posterior density interval (HPD) estimates. The mean mutation rate was estimated as 1.665 × 10^−3^ nucleotide substitutions per site per year. A constant rate of evolution is observed in the last decade.

**Figure 4:**
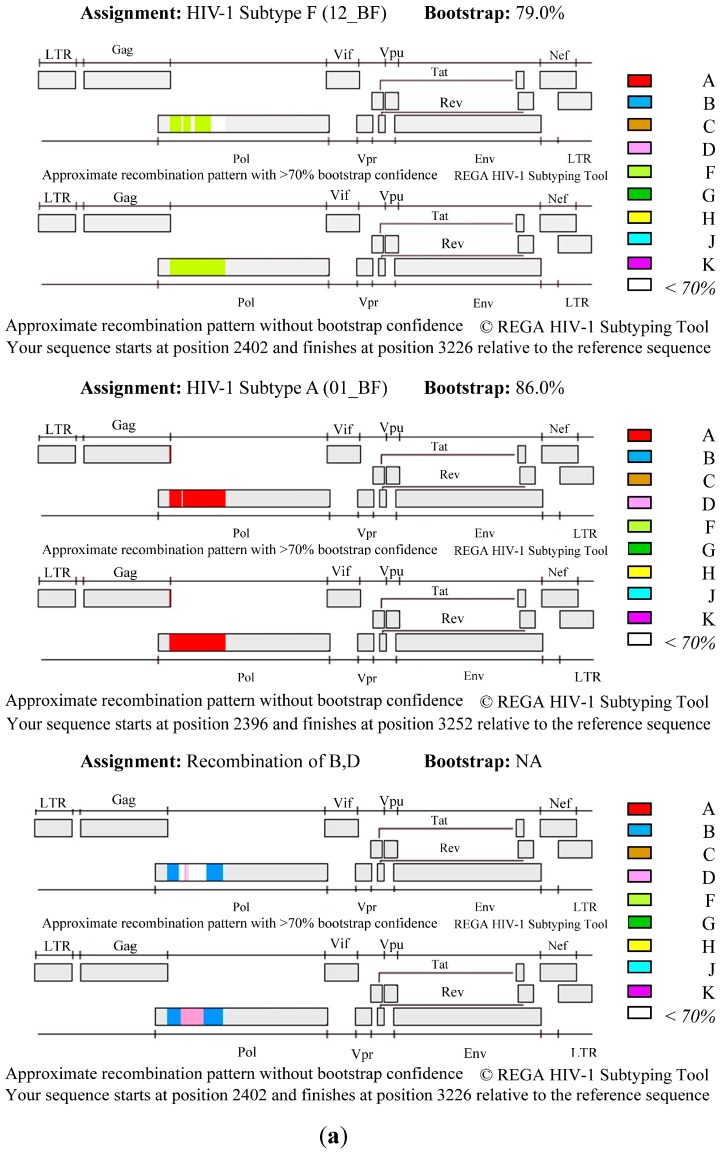

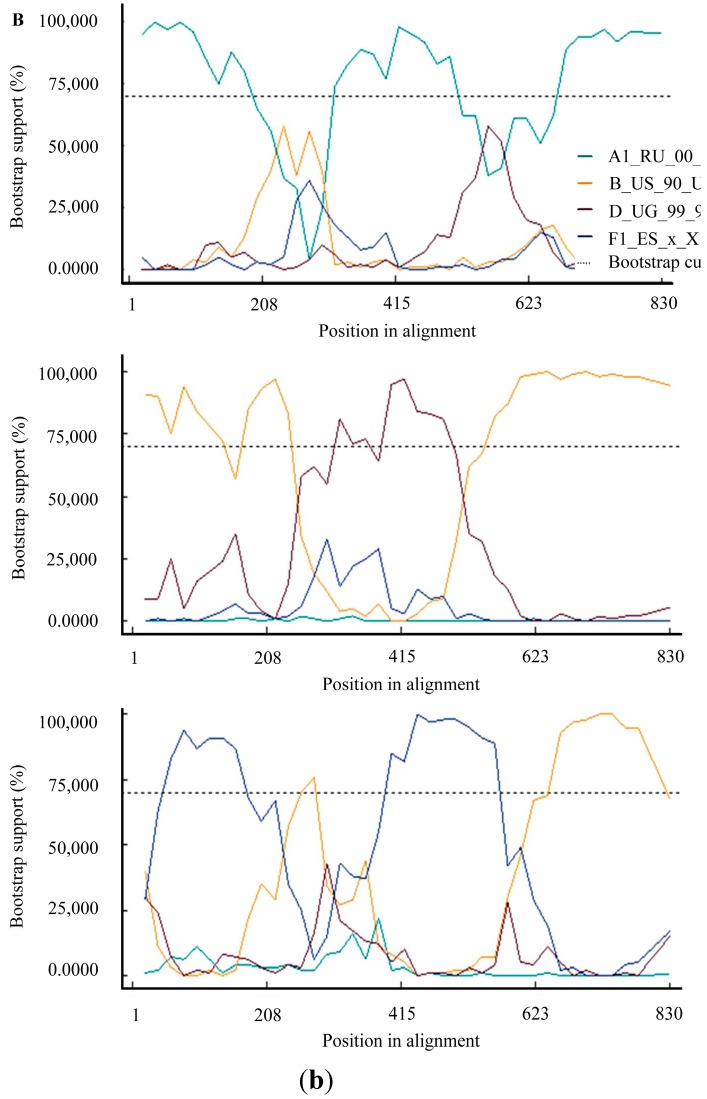
HIV-1 B/D recombination profiles. (**A**) Plots show a graphical representation of the HIV-1 genome showing the genomic region of the query sequence with the start and end positions related to the reference sequence (HXB2) for three examples of recombination profiles. The analyses were performed using REGA (version 3). (**B**) Graphical representation of the breakpoint coordinates as determined by RDP algorithm implemented by Recombination Detection Program v4.61. The reference sequences included were: EU345717, AY173953, D_UG_99_99UGG35093_A (REGA) and F1_ES_x_X1670_DQ9790 (REGA) [27].

As shown in Figure 1, the analysis revealed that HIV-1 subtype B was the main genetic form in Puerto Rico (90%), including a high prevalence of HIV1-B recombinants (in the given proportions): B/D (6%), D/B recombinant (0.6%), and B-like (3%). Nevertheless, our analysis also indicates that other subtypes, including A, and F, also present. A much smaller number of cases were found to have the following subtypes (in the given proportions): A1B (0.3%), BF1 (0.2%), subtype A (01-AE) (0.1%), subtype A (A2) (0.1%), subtype F (12BF) (0.1%), CRF-39 BF-like (0.1%), and others (0.1%). Some of the recombinants appeared as early as in 2001 (Figure 2). The BSP analysis provides new evidence that suggests that the B/D recombinants experienced a fast exponential grow from 2000 to 2005, followed by a decline in the growth rate in the last decade (Figure 3). The mean mutation rate was estimated to be 1.665 × 10^−3^ nucleotide substitutions per site per year. A constant rate of evolution is observed in the last decade. Analysis of the breakpoints in B/D recombinants using the Recombination Detection Program (version 4) [18], revealed that not all the B/D recombinants exhibit similar breakpoints (Figure 4; Figure S2), suggesting that the recombination was not generated by a unique event that later spread in the population.

Our analysis indicated the presence of other subtypes, including A, D and F. Non-B subtypes have thus far been identified only in the form of recombinants, but such recombinants were present in Puerto Rico as early as 2001. There was no indication of subtype C, which has been predominantly associated with heterosexual transmission in other parts of the world [19]. Nevertheless, the proportion of B/D recombinants is higher in our region than it is in others [20]. However, in order to improve the accuracy of our results, it is necessary to investigate additional sequences from that time period and other countries, which sequences are not currently available. Additional data should help more precisely define the impact of B/D recombination.

Recent analyses suggest that recombination can introduce or eliminate drug resistance mutations [21]. Recombination and drug resistance mutations may increase in the absence of close virological monitoring and because of poor access to alternate drugs (in case of virological failure) [22]. In the highly mobile world of today, it is important to contain the HIV evolution rate to the minimum so that the emergence of an epidemic caused by a highly unusual strain may be avoided. In addition, the implementation of appropriate patient-monitoring systems to assess drug-resistance mutations is an essential element of patient care.

The Bayesian analysis generated for our project provides new information indicating that the epidemic in Puerto Rico (B/D recombinants) experienced a deceleration in growth rate during the period spanning from 2005 to 2014. During that time, advances in treatment made combination therapy more convenient and effective for many patients, leading to incremental increases in treatment adherence [23,24]. For chronic diseases such as HIV, adherence to a given therapy is a challenge. Nevertheless, if patients increase adherence to their medication, viral evolution will be better controlled and the probability that the virus will not mutate or become resistant is going to be high [25]. In addition, ever since the first cases of AIDS on the island were reported, local health departments and community organizations have become highly involved in establishing prevention programs that provide care, health insurance coverage, and support. These initiatives encourage the population to seek an early diagnosis of HIV [8]. Adherence to ART and better health care provision from 2006 going forward may be responsible for slowing the rate of HIV evolution.

## 4. Conclusions

Overall, our data suggest that the *pol* gene in Puerto Rico has a high proportion of B/D recombination, despite the inter-subtype that was reported previously (inter-subtype B/F). The constant monitoring of virus evolution, the continuous development of prevention programs, and adherence to HIV treatment regimens are vital elements necessary for the control of the HIV-1B virus’s evolution, especially in the high-risk population found in Puerto Rico.

## Supplementary Files

Supplementary File 1
