# Supplementary material for: The Genetic Diversity and Evolution of HIV-1 Subtype B Epidemic in Puerto Rico
Source: Int J Environ Res Public Health. 2015 Dec 23;13(1):55. doi: 10.3390/ijerph13010055 (PMC4730446; doi:10.3390/ijerph13010055)
Supplement: Supplementary File 1 [file ijerph-13-00055-s001.pdf]

The Genetic Diversity and Evolution of HIV-1 Subtype B Epidemic in Puerto Rico

---

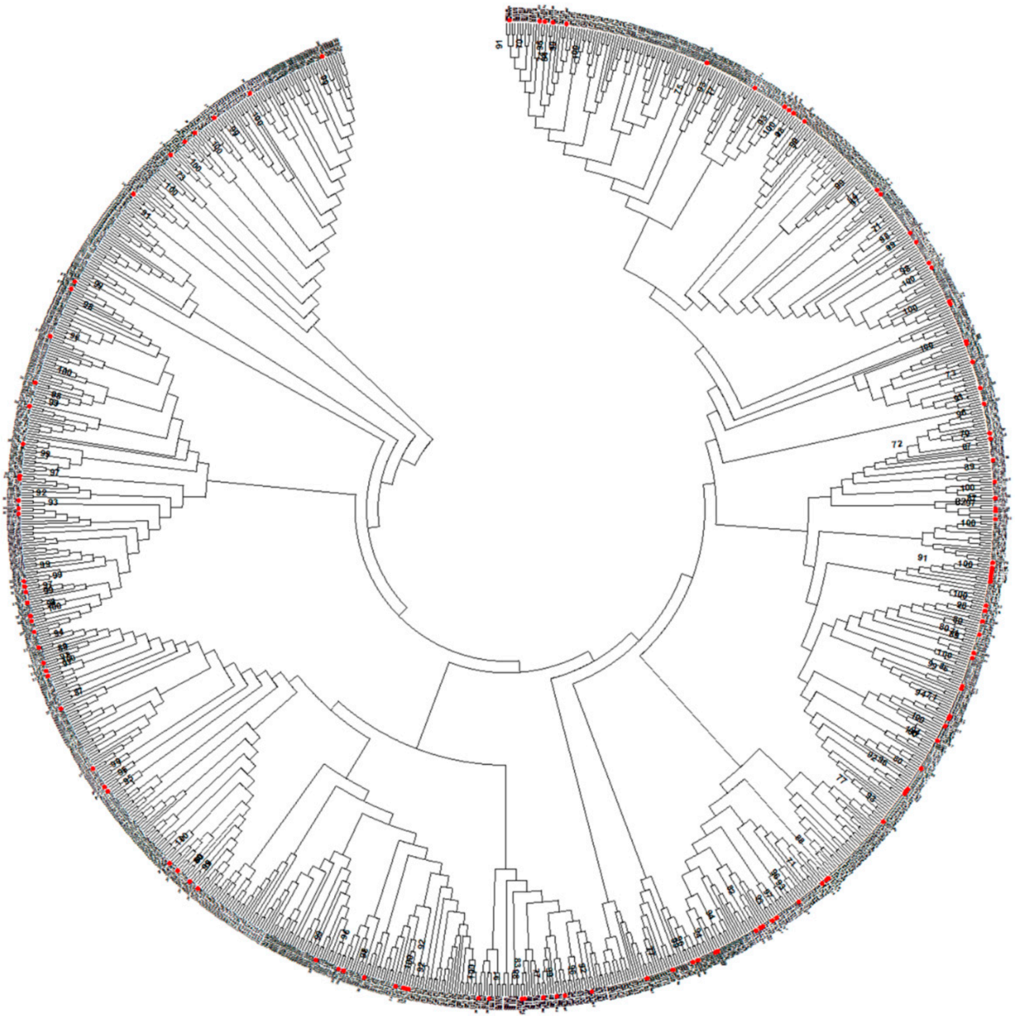

Figure S1. Sequences relationships were inferred by using the Maximum Likelihood method based on the Tamura-Nei model. The analyses were conducted in MEGA6. The tree with the highest log likelihood (−849,484.1105) is shown. The percentage of trees in which the associated taxa clustered together is shown next to the branches. If two sequences clustered together (bootstrap value for both sequences ≥0.7), one of the sequences was excluded from the analysis. Samples discarded are identified by bullet (•). Initial tree(s) for the heuristic search were obtained automatically by applying Neighbor-Join and BioNJ algorithms to a matrix of pairwise distances estimated using the Maximum Composite Likelihood (MCL) approach, and then selecting the topology with superior log likelihood value. A discrete Gamma distribution was used to model evolutionary rate differences among sites (5 categories (+G, parameter = 0.3378). The analysis involved 1071 nucleotide sequences.

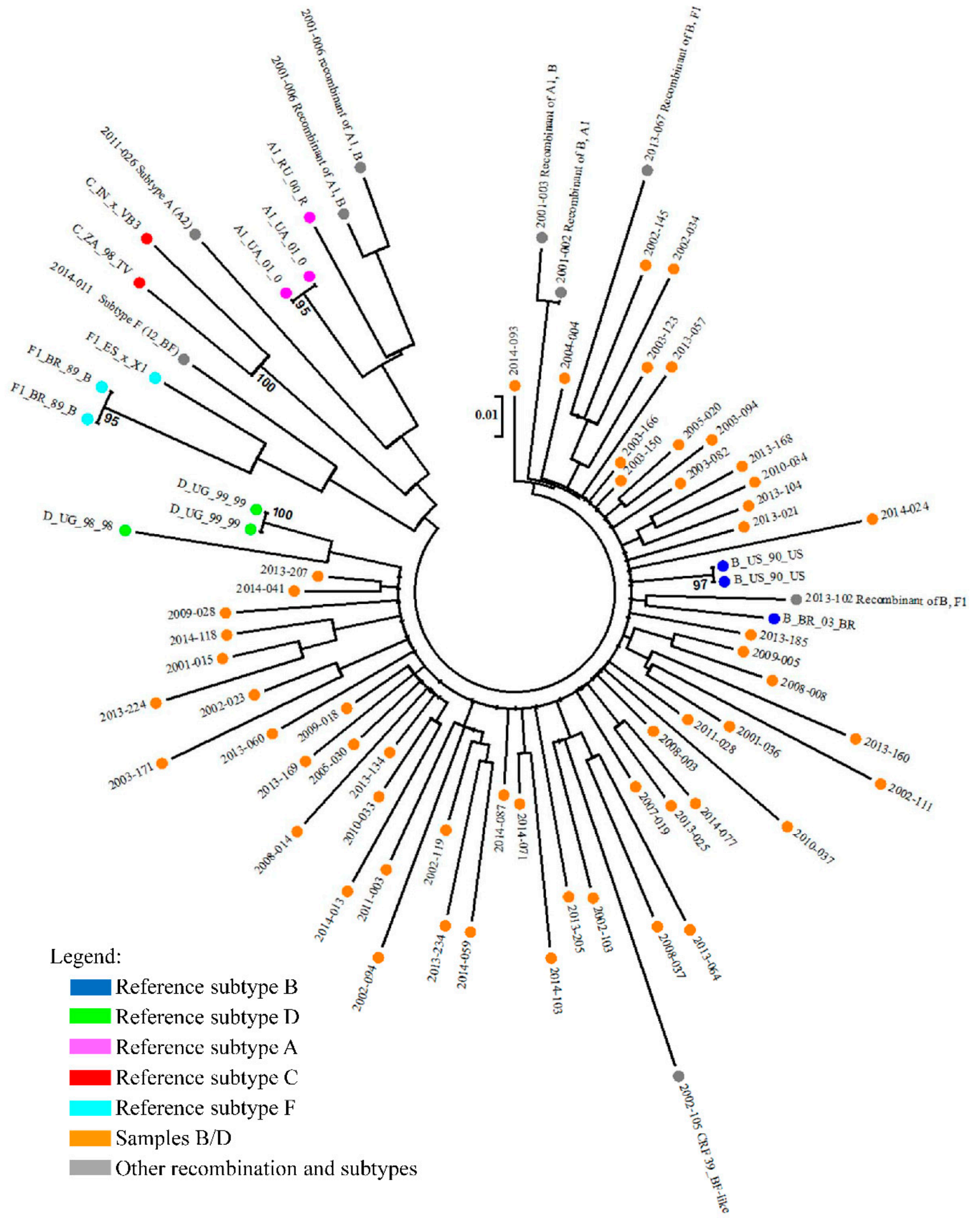

Figure S2. Sequences with recombination events were compared against reference sequences from REGA (version 3) to assess the recombinants relatedness. Maximum likelihood analysis of the sequences with recombination was performing by MEGA (v6).The tree with the highest log likelihood (−269,263.2199) is shown. The percentage of trees in which the associated taxa clustered together is shown next to the branches. The tree is drawn to scale, with branch lengths measured in the number of substitutions per site. There were a total of 723 positions in the final dataset. The colors indicate the subtype assignment by REGA (v3). The references sequences were obtained by REGA (v3).

© 2015 by the authors; licensee MDPI, Basel, Switzerland. This article is an open access article distributed under the terms and conditions of the Creative Commons Attribution license (http://creativecommons.org/licenses/by/4.0/).
